# A demographic comparison of NASA astronauts and commercial spaceflight participants

**DOI:** 10.1038/s41526-025-00559-9

**Published:** 2026-01-15

**Authors:** Katie J. Hogan, Emmanuel Urquieta, Robert J. Reynolds

**Affiliations:** 1 Baylor College of Medicine, Houston, TX USA; 2 University of Central Florida College of Medicine, Department of Medicine, Orlando, FL USA; 3 Mortality Research & Consulting, Inc., Houston, TX USA

**Keywords:** Business, Industry, Technology, Aerospace engineering, Economics, Government, Interdisciplinary studies

## Abstract

The accelerated pace of commercial spaceflight is rapidly increasing the number of civilian spaceflight participants (SFP). In this study, SFPs were shown to be older with a broader age range than NASA astronauts, and have more diverse educational backgrounds and occupations. K-means clustering using demographic data showed that NASA astronauts fall broadly into categories reflecting military pilot training versus non-pilots, often scientists and engineers. Conversely, SFPs fall into five categories we label as Professional Astronauts, Veteran Adventurers, Young Adventurers, Celebrities, and Science Tourists. Overall, these SFPs are a more diverse and globally representative population than traditional governmental astronauts, and the demographics of each cohort directly reflect the mission objectives of their respective agencies and companies. Demographics provide insight into the goals and priorities of both governmental and commercial spaceflight organizations, and further investigation of these trends will be important as human spaceflight continues to change in the decades to come.

## Introduction

Space exploration has continually captivated humanity, with governmental space agencies at the forefront of exploring the final frontier. However, with the advent of commercial spaceflight, people traveling to space no longer have the same homogeneity of background that has been the hallmark of government-trained astronauts. Marked differences would be expected between government astronauts, who are highly selected from a medical and psychological perspective and trained by national space agencies, and commercial spaceflight participants, or SFPs, who are largely self-selected from the private sector^1^. The demographics of these two groups—ranging from age and education to sex and professional experience—offer unique insights into how different pathways to space are evolving and being rapidly developed. Understanding these differences allows us to reflect on the broader trends in the spaceflight industry and, thus, the changing needs of space medicine.

The selection processes for government astronauts and commercial SFPs differ significantly. Government astronauts are typically selected by national space agencies such as the United States National Aeronautics and Space Administration (NASA) or Roscosmos (Russian Federation), as well as NASA partner agencies like the European Space Agency (ESA), Canadian Space Agency (CSA), and the Japan Aerospace Exploration Agency (JAXA), and recently by the China National Space Administration (CNSA). Governmental astronauts are carefully chosen through a rigorous selection process that focuses on specific qualifications, including education and experience, physical and medical fitness, and psychological makeup^2^. For example, recent astronaut candidate selections have required advanced degrees (master’s or doctoral) in fields like engineering, physical sciences, mathematics, or medicine, and are expected to have several years of professional experience in their discipline^3,4^. Though no longer required, many governmental astronauts have military backgrounds as well. In contrast, commercial spaceflight has no standard selection criteria, although some recent efforts have looked at developing medical screening recommendations^2,5^. As most commercial SFPs are customers rather than employees, there are typically few, if any, educational or vocational requirements beyond the ability to acquire a seat, via purchase or lottery, and be medically cleared by each of the commercial spaceflight providers^6,7^.

Governmental astronauts typically must pass a rigorous physical examination and undergo psychological testing as part of both initial selection and ongoing fitness for duty. This includes vision standards, cardiovascular health, and the ability to handle stressful environments and situations, among others^3,4,8,9^. When commercial companies contract with NASA, they must adhere to NASA standards. However, commercial spaceflight companies may set their own medical standards according to their individual risk tolerance for flights not performed under contract with NASA, which has led to the inclusion of individuals with health conditions that may have been previously excluded from spaceflight, such as one passenger with a prosthetic knee^6,10^.

A further difference between governmental astronauts and SFPs is in their training requirements. Once selected, governmental astronauts undergo years of intensive training as astronaut candidates, which includes extravehicular activity simulation, survival training, physical conditioning, and learning to operate spacecraft systems and robotics^11,12^. While commercial spaceflight companies have instituted both medical screening and pre-flight training programs, most components are flexible and take place over weeks to months, depending on the mission design and profile. Overall, training remains far less intensive than the training required by governmental space agencies^6,10,13,14^. This reduced and condensed training is likely due to the fact that most commercial SFPs are acting as passengers and therefore not active participants in piloting or mission execution.

Both pathways reflect the evolving nature of human spaceflight, with government astronauts embodying the technical rigor and long-term scientific objectives of national space agencies, while SFPs represent the expanding democratization and commercialization of space travel. As of 2026, the number of SFPs is steadily growing, driven primarily by the rise of commercial spaceflight companies like SpaceX, Blue Origin, Virgin Galactic, and Axiom Space^15^. Although civilian astronauts are still a minority compared to government astronauts, several milestones have significantly increased their numbers in recent years.

Space tourism has seen rapid growth in the 2020 s alongside the development and maturation of additional commercial spaceflight companies. The industry and number of manned missions have increased exponentially since 2021 with the introduction of commercial suborbital flights by companies like Blue Origin and Virgin Galactic, allowing civilians like Jeff Bezos, William Shatner, and other SFPs to briefly experience space^3^. SpaceX increased the number of SFPs with its Inspiration4 mission in 2021, where four SFPs orbited Earth, including the first all-civilian crew^16^. Likewise, Axiom Space has launched private astronaut missions (PAM) to the ISS, working closely with NASA through the PAM program^17,18^. These PAM missions have enabled the development of guidelines and recommendations to operate jointly between government astronauts and SFPs^19^. In addition, when hiring crew for active piloting or customer service, commercial spaceflight companies commonly employ former astronauts or highly trained pilots with military backgrounds. This practice results in an additional area of overlap and cooperation between governmental and commercial spaceflight agencies.

While conflicting definitions for the boundary of space exist, NASA and the US Air Force define space as any altitude above 80 km (50 mi), and this standard is used primarily for awarding astronaut wings to personnel^20^. Using this standard, as of 2024, ~100 civilian astronauts have flown in space, including SFPs and private astronauts involved in scientific or PAM missions. This figure is expected to rise sharply with increasing access to commercial space travel, as companies aim to lower the cost of spaceflights and launch more frequent missions. The number could reach the hundreds within the next decade as space tourism and private space exploration mature.

The differences in selection processes, training requirements, and SFP background signify that further differences in demographics and characteristics may be present. Quantifying and analyzing these differences is important as the expanding demographics of those participating in spaceflight undoubtedly stretch the boundaries of what is known regarding human physiology in space. As organizations and regulatory bodies attempt to understand the limits that must be put in place for human health in spaceflight and the additional studies needed to further characterize important health attributes, an initial survey of commercial SFPs in comparison to baseline data about NASA astronauts is warranted. Additionally, a well-formed concept of the types of people participating in commercial spaceflight allows commercial spaceflight companies and NASA to identify potential areas of cooperation and align their interests and missions.

This study aims to characterize the demographics and characteristics of these commercial SFPs and contrast this population to NASA astronauts in terms of sex, age, education, and occupation. Additionally, via k-means clustering, both NASA astronauts and SFPs have been broken down into sub-cohorts with similar characteristics based on sex, education, military training, pilot certification, and year of launch, enabling a more nuanced exploration of the types of individuals who have taken part in commercial space flight to date. The current study provides new information and a classification of SFPs. It documents the diversity of this rapidly expanding population—diversity which corresponds to the goals and ambitions of commercial spaceflight companies and may enable a more broadly representative study of human physiology and capabilities in space.

## Results

SFPs were defined as individuals, both passengers and crew, who participated in a commercial, non-governmental spaceflight, as defined by the FAA, through November 15, 2024. Demographic data for 102 SFPs were gathered and first directly compared to an existing demographic database of NASA astronauts (see Methods).

### Female participation in spaceflight

Owing largely to the history of NASA’s selection criteria for astronauts, only 16% of the astronauts selected to the US Astronaut Corps have been female. In its first seven classes (1959–1969), NASA selected 73 astronauts, 100% of whom were male. These astronauts were selected mainly from pools of military jet pilots, though the agency also selected two smaller groups of doctoral-level scientists in this period. The first 6 female astronauts were selected in 1978, as part of a class of 35 new astronauts who would be the first to fly the Space Shuttle. After 1978, at least 1 female was selected in each astronaut class, and overall, females have composed 20% of the astronauts selected since that time.

Commercial space flight missions have completed suborbital flights, flights to LEO, and flights to the ISS, with widely varying mission durations (Table 1). Commercial spaceflight began in 2001, when the first “space tourist” visited the ISS on a Soyuz spacecraft. From 2001 to 2009, there were seven such flights, and all but one of the space tourists were males (85.7% males). Since 2019, when dedicated spaceflight companies began flying multi-participant crews, 23 out of 95 SFPs have been female. This means that of the 102 SFPs since 2001, 24 of them (24%) have been female.

**Table 1:** Included commercial space flights and select characteristics

Company	Destination	Mission	Year	Duration	SFPs
Space adventures	LEO/ISS	ISS EP-1	2001	7 d	1
		ISS EP-2	2002	8 d	1
		ISS EP-3	2005	9 d	1
		ISS EP-4	2006	12 d	1
		ISS EP-12	2007	10 d	1
		ISS EP-13	2008	12 d	1
		ISS EP-14	2009	14 d	1
		ISS EP-15	2009	11 d	1
		ISS EP-20	2021	14 d	2
Axiom space	LEO/ISS	Ax-1	2022	17 d	4
		Ax-2	2023	10 d	4
		Ax-3	2024	21 d	2
SpaceX	LEO	Inspiration4	2021	3 d	4
	LEO	Polaris Dawn	2024	4 d	4
Virgin Galactic	Suborbital	VF-01	2019	15 min	3
		VF-03	2021	15 min	1
		Unity 22	2021	15 min	3
		Galactic 01	2023	15 min	4
		Galactic 02	2023	15 min	4
		Galactic 03	2023	15 min	3
		Galactic 04	2023	15 min	3
		Galactic 05	2023	15 min	3
		Galactic 06	2024	15 min	4
		Galactic 07	2024	15 min	6
Blue Origin	Suborbital	NS-16	2021	10 min	4
		NS-18	2021	10 min	4
		NS-19	2022	10 min	
		NS-20	2022	10 min	6
		NS-21	2022	10 min	6
		NS-22	2022	10 min	5
		NS-25	2024	10 min	6
		NS-26	2024	10 min	6

### Age at selection and first flight

**Fig. 1:**
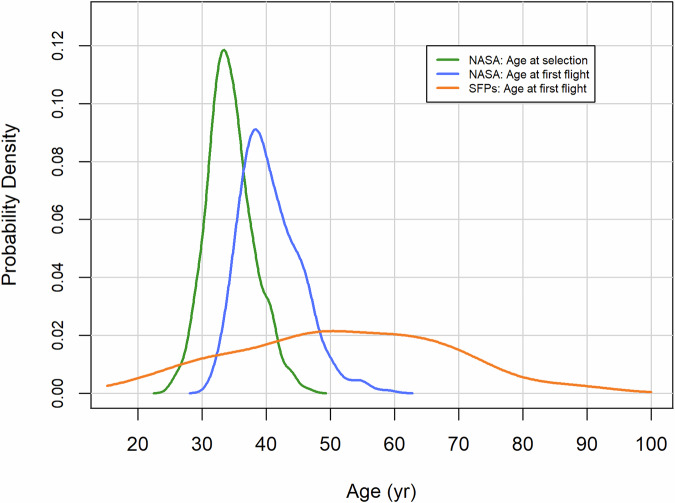
Distribution of age at selection and first flight for NASA astronauts (green and blue), and age at first flight for commercial spaceflight participants (orange). NASA astronauts are selected within a relatively narrow age range (26‒47 yr), then, after a minimum training period, complete their first flight between 2 and 19 years later. Commercial astronauts have had a much wider age range (18‒90 yr) and typically have only months between being announced as a spaceflight participant and going to space.

As seen in Fig. 1, astronauts in the NASA Astronaut Corps have ranged between 25.2 yr and 46.5 yr of age at time of selection, with a median of 33.9 yr (MAD = 3.2 yr). Owing to the extensive 2-year training period before flight eligibility, waiting times for mission assignment, and additional training after mission assignment, the median age at first flight for NASA astronauts is ~5 years older than that at selection, at a median of 39.8 yr (MAD = 4.4 yr). The range of age at first flight is also increased by 5 years, with values between 32.1 yr and 58.8 yr. Though this shift in the age distribution between age at selection and age at first flight is at least 2 years in all cases, the gap between selection and first flight has been as long as 18–19 years for some astronauts, with a median waiting period of 5.6 years. A Wilcoxon rank-sum test confirms that the difference in distributions is large enough to be statistically significant (*W* = 93256; *p* < 0.0001).

The range of age at flight for the commercial astronauts is considerably wider, and overall, commercial spaceflight participants tend to be older than NASA astronauts, with a greater variance in age. The range of age at first flight among commercial spaceflight participants encompasses both the youngest person to reach space (18 yr) as well as the oldest (90 yr). Among the 96 commercial participants whose ages were known to us, the median age was 51 yr (MAD = 17.8 yr). This median is 11 years higher than that of the NASA astronauts, with a standard deviation that is more than three times larger. Figure 1 shows kernel density plots for these age distributions.

Shapiro–Wilk tests for normality indicated that the commercial spaceflight participant age distribution follows a normal distribution (*W* = 0.989; *p* = 0.5922), while the skew toward older ages means that the age at first flight for NASA astronauts does not (*W* = 0.961; *p* < 0.0001). As the two distributions differed substantially in both shape and central tendency, no further statistical testing was performed.

### Education and occupation

Though serving as a NASA astronaut is a full-time occupation, it is not the astronauts’ first occupation. Likewise, commercial spaceflight participants have careers separate from their spaceflight activities, since most are not professional astronauts. The occupations of astronauts and commercial spaceflight participants were therefore examined to garner insight into their training and skills.

Marginally, 70% of the Astronaut Corps have a history of military service, and 69% are certified pilots. The overlap in these characteristics was not complete, however, as only 88% of astronauts who had a history of military service were pilots. Taken together, 61% of the overall NASA astronaut cohort had a history of military service and were simultaneously certified pilots. This leaves 8% of the cohort that are certified pilots with no history of military service, 9% with a history of military service but who were not certified pilots, and 22% that had neither a history of military service nor were certified pilots.

Only 19% (19/102) of the commercial spaceflight participant group had a history of military service. However, 39% of the group were trained pilots (40/102). While many of these pilots were private citizens, 14 had specific training as military pilots. Six of these former military pilots were commercial spaceflight crew, meaning they were employees paid to pilot spacecraft on commercial space flights.

**Fig. 2:**
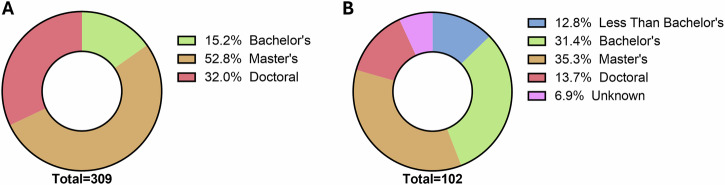
Educational attainment of NASA astronauts and commercial spaceflight participants. **A** NASA astronauts and **B** commercial spaceflight participants. As demonstrated by these donut charts, the majority of NASA astronauts have a master's degree, with doctoral degrees occurringtwice as often as bachelor's degrees. SFPs are twice as likely to have bachelor's degrees as doctoral degrees and have a wider range of educational attainment overall, including almost 13% with less than a bachelor's degree.

No NASA astronauts had less than a bachelor’s degree (Fig. 2). Overall, about 15% of astronauts had a bachelor’s degree, 53% had a master’s degree, and 32% had a doctoral-level degree (MD, PhD). In keeping with the non-professional and more inclusive nature of commercial spaceflight, educational attainment among commercial spaceflight participants is more variable than among astronauts and generally lower. Among the 95 participants whose educational attainment was known to us, just under a third of participants had either less than a bachelor’s degree (13%) or a doctoral degree (14%), about a third had a bachelor’s degree (31%), and about a third had a master’s degree (35%).

**Fig. 3:**
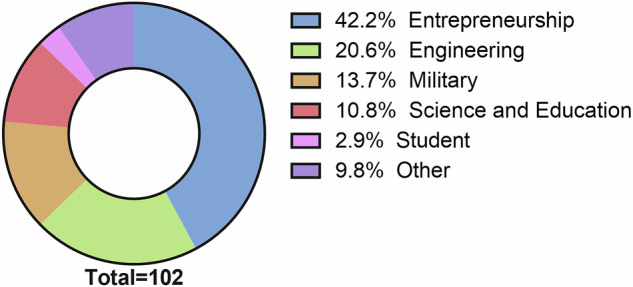
Relative frequency of occupations for SFPs. The plurality of SFPs have been entrepreneurs or businesspeople, followed by engineers.

Due again to the different selection mechanisms of commercial spaceflight participants, the occupational background of commercial astronauts is broader than that of NASA astronauts (Fig. 3). While the majority are either employed by commercial spaceflight companies or are paying customers, several seats on commercial space flights have been auctioned off or donated to charities, meaning that a more diverse range of socioeconomic backgrounds are represented than might be expected were all seats self-funded. Most commonly, commercial spaceflight participants have come from entrepreneurship backgrounds (42%), which here includes entrepreneurs, executives, and investors. Engineers (21%) as well as scientists, educators, and physicians (Science and Education, 11%), were also well-represented within the commercial cohort, reflecting some overlap in expertise with NASA astronauts. Among commercial crews, individuals were most likely to have a military occupation (75%), while the remaining 25% were engineers. Additionally, less well-established occupations of commercial spaceflight passengers falling within the ‘Other’ category included adventurers, explorers, and film producers. The broader age range of commercial spaceflight participants meant some younger passengers were also enrolled as full-time college students. Additionally, two passengers were identified primarily as internet content creators, which created opportunities for exposure to and education about space exploration for a wider audience.

### Nationality

While the companies in this study are based out of the US and the UK, commercial spaceflight also engages individuals from countries that might not otherwise be able to participate in crewed space missions. The US remained the most common nation of origin even for commercial spaceflight participants (52.9%), not including those with dual US citizenship. Overall, commercial missions have included individuals with citizenship in 24 different nations in total when accounting for dual citizenship.

### NASA demographic patterns

Next, groups of NASA astronauts were segmented using k-means clustering, a type of unsupervised machine learning that groups observations in an attempt to minimize the within-group variances and maximize the between-group variance. The number of groups (clusters) is designated by the analyst, and examination of a scree plot is commonly used as a guide for this process. The scree plot for the NASA cohort (not shown here) suggested four groups would optimally partition the data. In so doing, the clustering algorithm divided the astronaut cohort by sex and subdivided the males by occupation, age, and year of selection. The patterns that emerged suggested that the male astronauts had been grouped into two groups of military pilots and a single group of (mostly non-military) mission technical experts. While these groupings created clear and meaningful patterns among the male astronauts, the female group seemed to be partitioned on sex alone. Closer examination suggested that within the group of female astronauts, the patterns of military pilots and non-military mission technical experts could also likely be extracted. The clustering algorithm was therefore run again, excluding sex as a clustering variable.

**Fig. 4:**
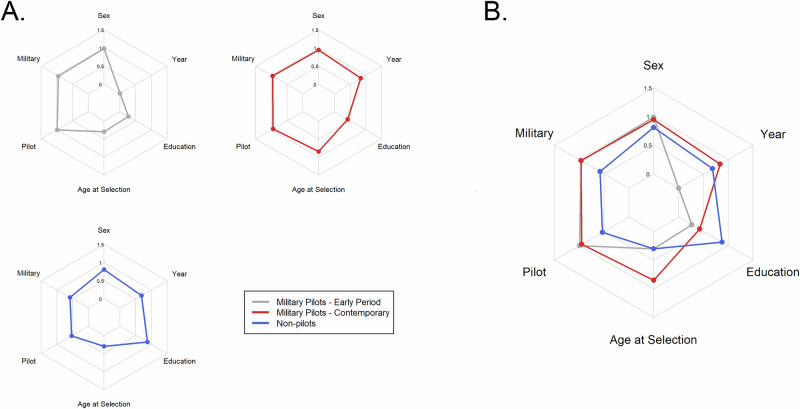
Polar plots for demographic factors within NASA astronaut groups defined by k-means clustering. **A** shows each group’s scores separately, while **B** combines them on a common grid. Values of variables are either prevalence (sex, military, pilot) or values scaled to a normal distribution with mean = 0 and SD = 1 (age, education, year).

When omitting sex as a clustering variable, the scree plot (see Fig. S1 in Supplemental Materials) this time suggested that three groups as an optimal solution, as it would balance group interpretability with group segregation. As suspected, the 3-group solution largely recreated the patterns seen previously and distributed the female astronauts into those groups as appropriate. The summary data for the 3 groups are presented in Table 2, and distributional comparisons are plotted in Fig. 4 as polar plots.

**Table 2:** Central tendency of demographic variables among groups formed from NASA astronauts

		Prevalence (%)						Median (MAD)		
	*n*	Male	Military	Pilot	Education			Year	Age at selection	Age at flight
**Group**					**BS**	**MS**	**Doctoral**			
Military pilots (early period)	122	97.5	90.9	99.1	31.1	51.6	17.2	1968 (11.1)	32.7 (2.0)	39.1 (3.3)
Military pilots (contemporary)	125	89.6	92.8	90.4	9.6	73.6	16.8	1996 (5.9)	36.9 (3.1)	42.3 (4.8)
Non-pilots	102	61.8	16.7	6.9	3.9	23.5	72.5	1992 (9.6)	33.0 (3.5)	38.7 (4.7)

The first two groups extracted from the astronaut cohort (gray and red lines in Fig. 4) were composed almost entirely of male astronauts who were pilots and had a history of military service. However, Wilcoxon tests (a non-parametric equivalent to the *t* test) indicated that age at selection (*W* = 2167.5; *p* < 0.0001) and age at first flight (*W* = 3448.5; *p* < 0.0001) were differentiators between these groups. Another key difference was the year of selection, where the first group tended toward earlier selection (median 1968) and the second group tended toward later selection (median 1996). A Wilcoxon test confirmed these differences are also large enough to be statistically significant (*W* = 404; *p* < 0.0001). Though they were somewhat similar in their educational attainment, a chi-square test confirmed that members of the more recently selected group were more likely to have a master’s degree and less likely to have only a bachelor’s degree (χ^2^(2) = 18.912; *p* < 0.0001). Given the high concentration of military pilots chosen, both of these groups were labeled as “Military Pilots,” but were differentiated by the additional labels of “Early Period” for the first group and “Contemporary” for the second.

The third group of NASA astronauts (blue lines in Fig. 4) was 62% male but nevertheless had the majority of the female astronauts (71%). In this group, only 17% had a history of military service, and only 7% were pilots. Overwhelmingly, the members of this group had a doctoral degree (73%), followed by terminal master’s degrees (23%), with only 4% having a bachelor’s degree. The group had a median age at selection of 38.7 yr, and though the group included astronauts selected between 1967 and 2017, the median year of selection was 1992. As occupation and educational attainment were the primary differentiators between this group and the other two, we identified this group as “Non-pilots”.

Further statistical testing highlighted the differentiating factors between the groups. Wilcoxon tests of the Non-pilots against the early period pilots revealed that there was no difference in age at selection (*W* = 6645.5; *p* = 0.3812) or age at first flight (*W* = 5490.0; *p* = 0.1852), but there was a significant difference in the year of selection (*W* = 1089.0; *p* < 0.0001). When comparing the Non-pilots group to Contemporary Pilots there were statistically significant differences in age at selection (*W* = 2562.5, *p* < 0.0001), age at first flight (*W* = 2594, *p* < 0.0001), and year of selection (*W* = 4746.5; *p* < 0.0001). Chi-square tests confirmed that the differences in proportions of educational attainment were also statistically significant between groups (χ^2^(2) = 263.09; *p* < 0.0001).

### SFP demographic patterns

**Fig. 5:**
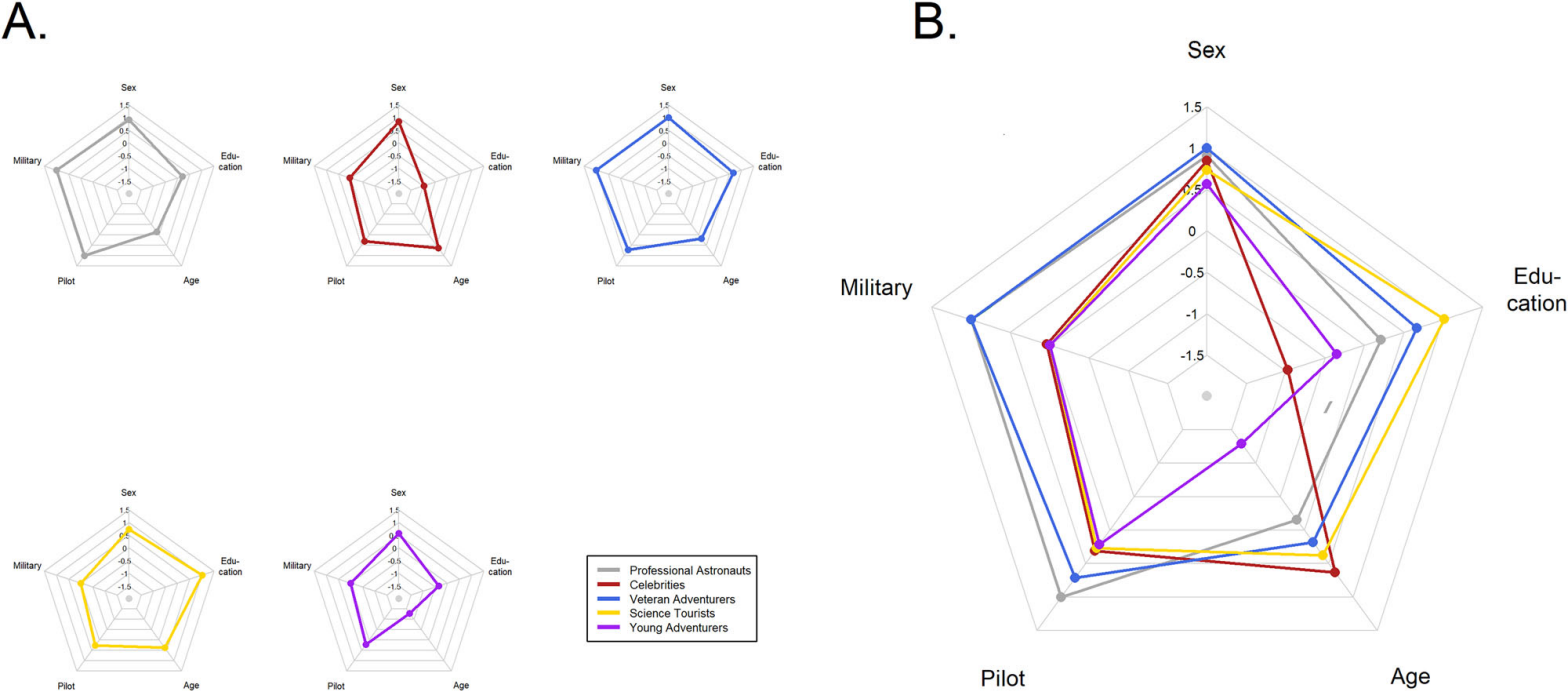
Polar plots for demographic factors within SFP groups defined by k-means clustering. **A** shows each group’s scores separately, while **B** combines them on a common grid. Values of variables are either prevalence (sex, military, pilot) or values scaled to a normal distribution with mean 0 and SD 1 (age, education, year).

To form demographic profiles among the SFPs, the same clustering procedure was employed as for the NASA astronauts. Due to missing information among the commercial SFPs, 93 observations were able to be used for the clustering. Five groups were created, as the scree plot (see Fig. S1 in the Supplemental Materials) suggested more than five would not lead to a meaningful further reduction in the within-group sum-of-squares. Additionally, we found five groups to be heuristically tractable. Following the results of the NASA clustering, sex was omitted from the clustering as we again did not want to form groups on the basis of sex alone. We clustered the SFPs on history of military service, history as a military pilot, and education. Table 3 shows the prevalence and central tendency of the final version of the five resulting groups, and Fig. 5 shows polar plots of their prevalences and scaled central tendencies. Nine SFPs could not be included in the analysis due to missing data.

**Table 3:** Central tendency of demographic variables among groups formed from commercial spaceflight participants

		Prevalence (%)							Median (MAD)	
					Education					
Group	*n*	Male	Military	Pilot	<BS	BS	MS	Doctoral	Year	Age at flight
Professional astronauts	11	90.9	100.0	100.0	0.0	27.3	72.7	0.0	2023.0 (1.48)	49.0 (14.82)
Celebrities	26	84.6	3.8	30.8	34.6	65.4	0.0	0.0	2022.0 (1.48)	60.5 (14.82)
Veteran adventurers	7	100.0	100.0	71.4	0.0	14.3	57.1	28.6	2022.0 (1.48)	52.0 (5.93)
Science tourists	26	73.1	0.0	26.9	0.0	0.0	53.8	46.2	2022.0 (1.48)	57.5 (12.60)
Young adventurers	23	56.5	0.0	21.7	17.4	43.5	39.1	0.0	2022.0 (1.48)	30.0 (5.93)

The first group (gray lines in Fig. 5) was moderately sized at 11 members and included only military pilots. The group was 91% male, with a median age of 49.0 yr. Seventy-three percent of the group had master’s degrees, and the other 27% had bachelor’s degrees. All of the participants in this group can be considered professional astronauts, as six are employees of Virgin Galactic (and considered crew under the FAA definitions), and five are astronauts with international governmental space agencies. Owing to this, this group was titled the “Professional Astronauts”.

The second group (red lines in Fig. 5) comprised 26 people, 85% of whom were males, with an overall median age at flight of 60.5 yr. Here, only one of the participants had a history of military service (Ed Dwight), though almost 31% had a pilot’s license. This group tended toward less education than other groups, as 35% had less than a bachelor’s degree and 65% had a bachelor’s degree. This group includes some of the principal owners of the commercial spaceflight companies, such as Jeff Bezos and Richard Branson, and celebrities such as William Shatner, Mark Bezos (brother to Jeff Bezos), Laura Shepard Churchley (daughter of NASA astronaut Alan Shepard), and Wally Funk (one of the “Mercury 13” female astronaut trainees of 1959). For this reason, this group was labeled the “Celebrities” group.

The third group (blue lines in Fig. 5) is a group of 7 males, all of whom had a history of military service. Five of them (71%) were pilots, and 5 were engineers and/or entrepreneurs in the aerospace industry. These SFPs had a median age at flight of 52.0 yr, and with comparatively low variability (MAD = 5.93 yr). Educational attainment for these SFPs tended toward master’s degrees (4/7 or 57%), though one member had a bachelor’s degree and two had doctoral degrees (14% and 29%, respectively). Owing to these characteristics, this group was dubbed the “Veteran Adventurers” group.

The fourth group (yellow lines in Fig. 5) consisted exclusively of people who had a master’s or doctoral degree. The group of 26 individuals was 73% male, with no history of military service among its members. However, about a quarter of the group (27%) had pilot licenses. The occupations of individuals in this group were primarily scientists, engineers, and educators (58%), though entrepreneurs were also well-represented (42%). Among those who were entrepreneurs, several were involved in New Space companies and/or venture capital firms for the same. The median age in this group was 57.5 yr. As this group’s essential characteristic is level of education and involvement in science and science entrepreneurship, this group was designated to be “Science Tourists”.

The fifth group (purple lines in Fig. 5) consisted of 23 participants, 57% of whom were males. While none of the members of this group had a history of military service, 22% held pilot’s certifications. While 4 participants (17%) in this group had less than a bachelor’s degree, 10 (44%) had a bachelor’s degree, and the remaining 9 (39%) had master’s degrees. This group was clearly the youngest, with a median age of 30.0 yr and an age range of 18 to 41 yr. As many of the members of this group were self-funded for their flights, this group was labeled simply “Young Adventurers”.

We manually reassigned three SFPs that were originally grouped into the Professional Astronauts cluster. We reassigned Eytan Stribbe and Scott Poteet to the Veteran Adventurers cluster, and reassigned Ed Dwight to the Celebrities cluster. Though these three SFPs had demographic characteristics that made them a good fit for the Professional Astronauts grouping, they were not professional crew or governmental astronauts, and therefore conceptually were a better fit in other groups. The data in Table 3 and Fig. 5 reflect the groups after these reassignments.

Testing the central tendencies of the demographic variables between the groups of SFPs demonstrated some statistically significant differences. A Kruskal–Wallis test (a non-parametric equivalent to ANOVA) for age demonstrated overall differences in the ages of the groups (χ^2^(4) = 54.872; *p* < 0.0001), and pairwise Wilcoxon tests revealed that this result was driven entirely by the Young Adventurers, who were significantly younger than all other groups. Chi-square testing demonstrated an overall difference in the distribution of pilots between clusters (χ^2^(4) = 25.581; *p* < 0.0001) as well as educational attainment (χ^2^(12) = 75.045; *p* < 0.0001). As sex was not a variable included in the clustering, we would not necessarily expect the clusters to have sex differences. Nevertheless, a chi-square test confirmed that the imbalances in sex between clusters were of marginal statistical significance (χ^2^(4) = 9.6038; *p* = 0.0477).

As shown in Table 4, a breakdown of SFPs by group membership and commercial spaceflight company reveals trends in the types of SFP each company has included as passengers and crew. First, only Virgin Galactic and Axiom Space flew the Professional Astronauts, as only Virgin Galactic employs commercial crew, and governmental astronauts all flew with one of these two companies. While Science Tourists and Young Adventurers have been present on flights from all companies, the greatest numbers of each have flown on Blue Origin and Virgin Galactic missions. The majority of Celebrities (62%) were Blue Origin passengers.

**Table 4:** Commercial spaceflight participant by cluster and commercial spaceflight company

Spaceflight company	Participant demographic group					
	Professional astronauts	Celebrities	Veteran adventurers	Science tourists	Young adventurers	Total
Axiom space	4	2	1	2	1	10
Blue origin	0	16	3	11	10	40
Space adventures	0	3	0	3	3	9
SpaceX	0	0	2	1	4	7
Virgin galactic	7	5	1	9	5	27
Total	11	26	7	26	23	93

## Discussion

The differences between NASA astronauts and SFPs can be easily observed in simple marginal distributions and point to the differential selection processes for governmental versus commercial spaceflight participants. For example, overall, there have been proportionately more female SFPs than female NASA astronauts, even in the period since 1978, wherein NASA’s selection rate for females increased. This relatively low inclusion of women in NASA missions is also seen for international astronauts and cosmonauts trained and launched by other governmental space agencies^21,22^. As mentioned, longitudinal studies have shown an increase in the number of female governmental astronauts over time, particularly with the loosening of requirements for military background and test pilot training^21^. For instance, 20% of 129 NASA astronauts for ISS missions were women, highlighting the increasing inclusion of females in more recent decades^22^. When considering differences in representation by sex between governmental and commercial SFPs, it is important to consider that while NASA hires its astronauts with specific mission goals in mind, commercial spaceflight companies largely operate on a first-come, first-served, interest-based paradigm. This means that gender parity in commercial spaceflight may be produced via specific mechanisms and organized campaigns by the companies, but also relies heavily on market demand. Commercial spaceflight is accelerating female inclusion in spaceflight in part through missions intentionally incorporating higher proportions of females.

Similarly, there are clear differences between the age distributions, educational attainment, and occupational pursuits between NASA astronauts and SFPs. The SFPs are generally older, but a much larger age range is seen in commercial missions, with even teenagers participating. The median NASA astronaut age here is similar to the average presented by Smith et al., a study which reported demographics for governmental astronauts employed by NASA, ROSCOSMOS, the ESA, CNSA, CSA, and the JAXA from 1969 to 2020. The authors reported an average age at first mission of 39.8 ± 5.3 yr^21^. Interestingly, Smith et al. also demonstrated that astronaut age at first flight has increased over time, perhaps reflecting an emphasis on the selection of governmental astronaut candidates with more established careers. This shift in age gives a clue as to the changing priorities of governmental spaceflight agencies, while commercial spaceflight agencies may attract older individuals in large part due to the significant fees required for participation.

With regard to education, while NASA astronauts uniformly have college degrees, it is worth noting that several commercial spaceflight participants, including Richard Branson, the CEO of Virgin Galactic, have spoken about leaving formal education in favor of entrepreneurial pursuits. For NASA missions, education and experience have direct ties to mission goals with regard to technical and engineering expertise to participate in space vehicle launch and maintenance, as well as experiments aboard the ISS. Currently, there is no significant need for high educational attainment among commercial SFPs aboard flights as passengers, who differ from those hired as commercial spaceflight crew, whose educational background more closely aligns with that of NASA astronauts. In contrast to the military, engineering, and science backgrounds of NASA astronauts, business and entrepreneurship are the most common occupations seen for SFPs. This is at least in part a selection bias because of the need for many SFPs to self-fund their expeditions in an environment of high prices. However, the broad range of occupations among SFPs highlights again the diversity of individuals brought together by commercial spaceflight missions.

Commercial spaceflight SFPs represent a large number of countries, including nations that are relative newcomers to spaceflight, a fact related to both governmental space agency partnerships and alternate means of acquiring seats. Commercial spaceflight has been exploited as a method for astronauts from nations with historically smaller space programs, such as Turkey and Saudi Arabia, to reach space relatively quickly and at comparatively low cost. Countries such as Sweden and Italy have also purchased seats for their astronauts on commercial space flights in spite of their continuing membership in ESA^23,24^. We speculate that this is again to accelerate their spaceflight experience at relatively low cost rather than wait for an ESA slot on a NASA vehicle. Through charitable auctions of seats, smaller countries without funded space programs, such as Antigua and Barbuda, have sent citizens to space^25^. Commercial spaceflight, with broad economic motivations to expand globally, therefore represents an important tool for increasing the diversity of nations reaching space.

The inclusion of clustering on demographics makes the comparison between these groups more nuanced. For example, it is interesting to note that the Professional Astronauts group, though small, is remarkably similar to the two Military Pilots groups from the NASA cohort, especially the Contemporary Pilot group. This may indicate that, though the spacecraft and concepts of operation are new for commercial spaceflight, the commercial spaceflight companies may still look for some version of the “Right Stuff” when selecting crew. This intuition is further reinforced by noting that some of the commercial crew have previous experience as NASA astronauts. Employing commercial crew undoubtedly plays a dual role: positive marketing and publicity by drawing a parallel to traditional spaceflight, while reducing in-flight risk by using highly trained and experienced operators.

As the goals of NASA missions have changed over time, two distinct career patterns have emerged: pilots and scientists. The first two clusters of NASA astronauts we formed here (Early Period vs Contemporary Military Pilots) have the commonality of military service, with increased educational attainment differentiating them in the contemporary period. Contemporary pilots tend to have master’s degrees in engineering, enabling them to build and repair the ISS in addition to piloting the vehicle. Scientist astronauts are primarily individuals with PhDs and MDs who, rather than operate vehicles on spaceflights, perform myriad scientific experiments while in space. Yet they, too, perform EVAs to service the station.

The distribution of SFP types among the commercial spaceflight companies serves as a reminder of the purpose of these commercial missions. For example, the inclusion of the Celebrities serves to increase the visibility of space flights, particularly with the inclusion of space-associated celebrities such as William Shatner, Wally Funk, and Ed Dwight. Blue Origin, specifically, has utilized this strategy, with 16 of 26 customers falling into this category, demonstrating an intentional ploy to raise the company's profile via the recruitment of well-known passengers. On the other hand, the largely self-funded Veteran and Young Adventurers served to establish a business model for space tourism, representing key customer bases. Science Tourists, with higher educational attainment, also represent a consequential portion of the audience to which commercial spaceflight companies wish to pitch the idea of spaceflight: those with significant scientific interest and investment capital. This scientific background mirrors that of the NASA Non-pilots, pointing to the draw of space among those with scientific curiosity and expertise. Both Young Adventurers and Science Tourists have been heavily featured in flights from Blue Origin and Virgin Galactic, which have made moves to increase the number and volume of flights in recent years. In this early expansion phase of commercial spaceflight development, these groups signify essential pieces of company strategies and mission priorities, including advertisement through celebrity participation and charity raffles and recruitment of individuals most likely to pay for a chance to go to space.

Additionally, there were 9 individuals who could not be incorporated into groups due to incomplete demographic data. These include self-described adventurers such as John Goodwin, Timothy Nash, Nicolina Elrick, and Carol Schaller. Ken Baxter, a lifelong space enthusiast, alongside Gorgio Manenti, Irving Pergament, and Ephraim Rabin, could fall easily within the Science Tourist group, as could Pantaleone Carlucci, an engineer who flew on a Virgin Galactic flight as a researcher. That we should readily identify groups for these individuals is a testament to the generalizability of the group profiles.

While national origin provides important information, the understanding of SFP diversity provided by the current study is incomplete due to the lack of race/ethnicity information, as these data were not readily available for all commercial spaceflight participants. It is also worth noting that several of the Professional Astronauts group of commercial SFPs were previously NASA astronauts, recruited by commercial spaceflight companies to act as commercial mission leaders or crew. This means that some individuals are included within both cohorts, specifically within the Contemporary Pilot group of the NASA cohort and the Professional Astronaut group of the SFP cohort. NASA and SFP cohorts, therefore, may be more similar than we might expect had the commercial spaceflight companies developed completely independently of NASA influence. However, the profile of the Professional Astronauts grouping is not so unique that new individuals could not be recruited who meet the profile. Additionally, the fact that commercial spaceflight companies hire crew at all demonstrates that the profile itself is of value to the companies and would be filled with similar personnel even if no former NASA astronauts were available.

In addition to broadening the demographics of those participating in spaceflight, commercial spaceflight has also widened the health requirement parameters for spaceflight. One limitation to the current study is limited access to health data due to privacy reasons within these cohorts. While NASA astronauts undergo rigorous health screenings to ascertain peak health prior to selection and deployment, commercial spaceflight participants are determined primarily by price and enthusiasm. The rigor of health screenings and medical standards within a commercial spaceflight context has been debated at length by national and international organizations, including the Aerospace Medical Association, International Academy of Astronauts, and the Space Transportation Agency^26^. A general consensus has emphasized the need for extensive medical history taking at the time of flight application and immediately pre-flight, paired with a physical exam by a space medicine physician. Likewise, the Federal Aviation Administration (FAA) released a 2006 report with guidelines and recommendations for screening commercial spaceflight crews and passengers, highlighting screening for injuries or diseases that may result in an in-flight emergency or death, compromise the health of the passenger or other vehicle occupants, or interfere with safety procedures^27^. However, while the FAA requires a valid FAA Class II Medical Certificate for commercial spaceflight crew members of suborbital flights and a valid FAA Class I Medical Certificate for orbital flights with annual evaluations, there are no standing requirements for passenger medical certifications^28^.

The risks to the health of an individual are relative to the mission destination and duration, whether that be suborbital or orbital. Studies by Blue et al. use centrifuge analogs to assess the risk of acceleration exposures experienced during suborbital flight in patients with well-controlled medical conditions (cardiac disease, diabetes, hypertension, lung disease, and history of back and neck musculoskeletal injury or disease)^29,30^. These studies showed that age, sex, medical history, and medication use did not significantly impact tolerance of acceleration, assessed via hemodynamic monitoring paired with other objective and subjective measurements. Additionally, alongside these medical conditions, in-dwelling devices for management (insulin pumps, implantable pacemakers), and a history of prior surgery did not increase the risk of significant acute cardiac, cerebrovascular, or respiratory events. These studies provide evidence supporting the safety of suborbital flight participants with well-controlled medical conditions and reassuring companies wishing to serve a broader population than governmental agencies. While this provides reassurance regarding the acceleration forces of suborbital flights, the effects of microgravity still convey significant risk for both suborbital flight participants and, over a larger timeframe, orbital flight participants. For this reason, governmental agencies, including NASA, CSA, ESA, JAXA, and Roscosmos, developed medical evaluation procedures and standards for spaceflight participants flying to the ISS in 2007^31^.

Nonetheless, commercial spaceflight companies have made a statement by the inclusion of commercial spaceflight participants with significant health conditions. Notably, Jon Goodwin, a passenger on Virgin Galactic’s Galactic 02 mission, has been stated to have had a Parkinson’s diagnosis for several years, which did not interfere with his medical clearance^32^. Likewise, SpaceX’s Inspiration4 mission in low Earth orbit featured Hayley Arceneaux, a cancer survivor with an in-dwelling titanium prosthetic bone and joint^33^. The inclusion of these and other individuals with both simple and complex health conditions in commercial spaceflights will prove important to expand the international knowledge base concerning human physiology in space, which, up to now, has been limited to individuals without significant medical conditions. As commercial spaceflight continues in the future, further in-depth study of the effects of health conditions on human physiology in space will be essential to determine the safety and efficacy of participants.

Commercial spaceflight is rapidly changing and expanding, promising to deliver a larger and increasingly diverse set of SFPs to suborbital and LEO destinations. Here we have documented that, thus far, commercial spaceflight has begun to fulfill that goal. Commercial spaceflight has flown SFPs that are older than NASA astronauts on average, but also have greater variability, with much younger and much older SFPs. SFPs are so far more frequently female, and have a wider range of educational attainment and vocation, as NASA astronauts have had higher educational attainment on average and have more often been military pilots. Segmenting NASA astronauts and SFPs revealed some similar subgroups, but also revealed new space tourist groups among the SFPs. These results highlight key differences in the types of people who participate in commercial spaceflight compared to governmental astronauts, which in turn is reflective of the current mission objectives for space flights in each domain. As commercial spaceflight continues to mature, its objectives will undoubtedly shift, and with it the demographics of commercial SFPs. Continued investigation of demographic trends will be needed in the years to come to document these ongoing changes.

## Methods

### NASA astronaut data

Demographic data for NASA astronauts were obtained from an existing database of NASA astronauts, as previously described^34,35^. The dataset used here contained information up to and including NASA’s 22^nd^ training class, selected in 2017. The dataset contained details on pre-selection occupation, including history of military service, pilot certification, and branch of the service, if any. NASA career details included date of selection to the NASA Astronaut Corps, as well as date of first flight, with flights through June 30, 2018, included. Demographics included age, sex, and education.

### Commercial spaceflight participant population and data

The FAA defines a spaceflight participant as someone who travels into space but is not a professional astronaut. Commercial spaceflight participants evaluated here include individuals who traveled to space on flights chartered by commercial, non-governmental entities, and included both spaceflight crew and passengers. All SFPs under analysis here participated in flights that met the NASA and US Air Force standards for reaching the boundary of space, as Virgin Galactic suborbital flights reach an altitude between 85-88 km, and Blue Origin suborbital flights reach an altitude between 105 and 107 km. These participants were identified via official press releases from five commercial spaceflight companies: Space Adventures, Virgin Galactic, Blue Origin, SpaceX, and Axiom Space. The flights included those with mission destinations of suborbital space and low Earth orbit, including the International Space Station (ISS). The included flights started with the ISS EP-1 mission in 2001 and extended to commercial space flights through November 15, 2024. Data on commercial spaceflight participants were collected from publicly available sources on the internet, including press releases and biographies on the aforementioned company websites, news articles, biographies on the participants’ own websites, and popular fan websites such as Spacefacts.de, Supercluster.com, and WorldSpaceFlight.com.

Data collected included age at first flight, sex, occupation, nationality, and mission details. In the event that the age of an individual was known by year but not precisely by birthdate or recorded age at flight, the individual’s birthdate was assumed to be July 2nd, mid-year, of their birth year, according to previously published methods^36^. For commercial spaceflight participants the time between announcement of participants for a spaceflight and completion of the spaceflight can usually be measured in months rather than years. For this reason, and owing to limitations in the available data for commercial spaceflight participants, we treat the age at announcement and age at first flight as synonymous. Occupations were grouped into broad categories of related fields to aid in comparison to those of NASA astronauts. For those with dual citizenship, both citizenships were considered when calculating the number of countries represented in commercial spaceflight. Due to the experience and expertise of NASA astronauts, there are individuals who retired from NASA and now work or participate in commercial space flights, meaning that they are represented within both datasets, with data such as age relative to mission. Only those participants meeting FAA requirements for designation as crew, i.e., actively participating in spacecraft piloting or flight coordination, are designated as crew within the text. In the event of missing data, individuals were excluded from the respective analyses.

### Statistical analysis

All statistical analysis was performed using R statistical computing software (The R Project for Statistical Computing)^37^. Both R and GraphPad Prism 6.01 were used to create additional plots^38^. Continuous distributions were assessed for normality using the Shapiro-Wilk test. For the Shapiro–Wilk test, the null hypothesis is that the distribution of the sample is normally distributed. As such, a significant *p* value represents evidence for non-normality in the sample.

Central tendency was assessed via the median and median absolute deviation (MAD) rather than mean and standard deviation. Accordingly, we used the non-parametric Kruskal–Wallis test to assess differences in medians across multiple groups, and the Wilcoxon Rank-Sum test for pairwise comparisons of medians. Differences in the distribution of frequency across categories were indexed using the Chi-square test for independence. For all singular tests, the alpha level was set at 5%, meaning a two-sided *p* value of 0.05 or less was considered to be a statistically significant difference. Whenever multiple pairwise comparisons were made, the Bonferroni correction was used, dividing the alpha level by the number of comparisons.

### Unsupervised machine learning

K-means clustering was used on the available demographic variables in the two cohorts separately to partition individuals into groups. The resulting groups (“clusters”) are such that individuals contained within each group are more similar to each other than they are to individuals in the other groups. Since the number of groups is at the discretion of the analyst, a typical first step in such analyses is to examine a scree plot, which shows the sum of the within-group variance as a function of the number of groups. The objective was to balance the number of groups against the amount of variability in the data explained by them, such that the groups were conceptually distinct, but not superficially so.

## Supplementary information

Supplemental_Figures.

## Data Availability

The datasets used and/or analyzed during the current study are available as the Astronaut and Spaceflight Participant Archive (ASPA) at https://github.com/osmed-admin/aspa.
