# Supplementary material for: A demographic comparison of NASA astronauts and commercial spaceflight participants
Source: NPJ Microgravity. 2026 Jan 15;12:14. doi: 10.1038/s41526-025-00559-9 (PMC12894856; doi:10.1038/s41526-025-00559-9)
Supplement: Supplementary file 1 — Supplemental_Figures. [file 41526_2025_559_MOESM1_ESM.docx]

A Demographic Comparison of NASA Astronauts and Commercial Spaceflight...
Katie Hogan, Emmanuel Urquieta, Robert J Reynolds

npj Microgravity

3ca50c16-b7ed-4bb2-80ab-c3bb09dc724b

**Supplemental Figure**


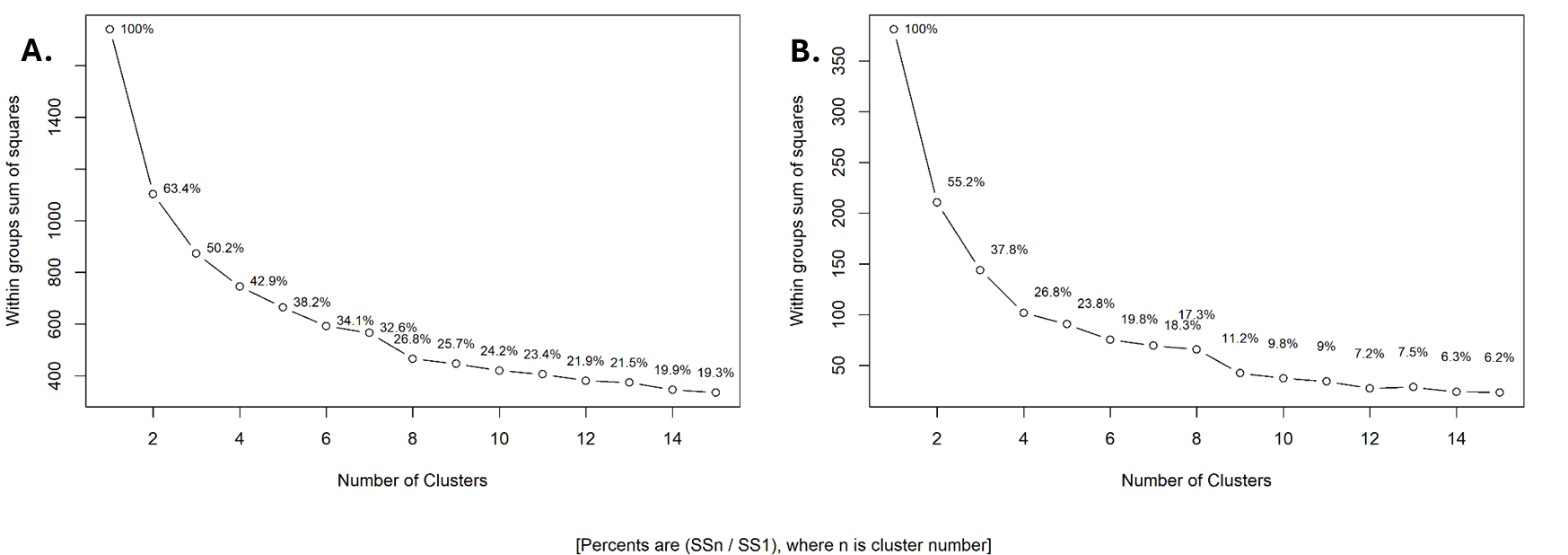


**Figure S1.** Scree plots produced via k-means clustering and development of (A) NASA and (B) commercial SFPs subgroups.
